# Proximal Femur Responses to Sequential Therapy With Abaloparatide Followed by Alendronate in Postmenopausal Women With Osteoporosis by 3D Modeling of Hip Dual‐Energy X‐Ray Absorptiometry (DXA)

**DOI:** 10.1002/jbm4.10612

**Published:** 2022-03-10

**Authors:** Renaud Winzenrieth, Paul Kostenuik, John Boxberger, Yamei Wang, Ludovic Humbert

**Affiliations:** ^1^ Galgo Medical Barcelona Spain; ^2^ Radius Health, Inc. Boston MA USA; ^3^ University of Michigan School of Dentistry Ann Arbor MI USA

**Keywords:** ANALYSIS/QUANTITATION OF BONE, BIOCHEMICAL MARKERS OF BONE TURNOVER, BONE MODELING AND REMODELING, DXA, OSTEOPOROSIS

## Abstract

Previous subgroup analyses from the ACTIVE trial in women with postmenopausal osteoporosis (NCT01343004) using three‐dimensional (3D)‐processing of dual X‐ray absorptiometry (DXA) scans indicated greater increases in total hip cortical volumetric bone mineral density (Ct.vBMD) and estimated indices of hip strength following 18 months of abaloparatide (ABL) versus placebo or teriparatide. The current post hoc analyses describe hip 3D‐DXA data for ACTIVExtend (NCT01657162), in which 18 months of ABL followed by 24 months of alendronate (ABL/ALN) increased hip and spine areal BMD (aBMD) and reduced fracture risk versus placebo (PBO) followed by ALN (PBO/ALN). In an ACTIVExtend subgroup (ABL/ALN, *n* = 204; PBO/ALN, *n* = 202), hip DXA scans retrospectively underwent 3D modeling via 3D‐Shaper software. Changes from baseline in cortical and trabecular compartments were calculated for total hip and hip subregions (femoral neck, trochanter, and shaft). Estimated strength indices comprising cross‐sectional moment of inertia, section modulus, and buckling ratio were calculated for each hip subregion. Correlations between bone turnover marker levels at the time of alendronate initiation and subsequent BMD gains with alendronate were also investigated within each group. Total hip trabecular and cortical 3D‐DXA parameters increased from baseline in both groups (all *p* < 0.001), with greater average increases for ABL/ALN versus PBO/ALN (trabecular vBMD: 10.87% versus 4.3%; cortical thickness: 2.32% versus 1.14%; Ct.vBMD: 3.41% versus 1.86%; cortical surface BMD: 5.82% versus 3.0%; all *p* < 0.001). Strength indices in the ABL/ALN group improved in all subregions versus baseline (all *p* < 0.0001) and versus PBO/ALN (all *p* < 0.02). In the ABL/ALN group, collagen type I N‐terminal propeptide (P1NP) levels at the time of alendronate initiation correlated with subsequent percent changes in all 3D‐DXA parameters with 24 months of alendronate therapy. In conclusion, sequential ABL/ALN or PBO/ALN treatment improves trabecular and cortical 3D‐DXA parameters at the hip, as well as strength indices of hip subregions, with greater increases with ABL/ALN versus PBO/ALN. © 2022 Radius Health, Inc. *JBMR Plus* published by Wiley Periodicals LLC on behalf of American Society for Bone and Mineral Research.

## Graphical abstract

Mid‐coronal sections of the hip depict mean changes in volumetric BMD. Greater increases in cortical regions are shown by more green color for participants who received sequential therapy with abaloparatide followed by alendronate versus those who received placebo followed by alendronate.

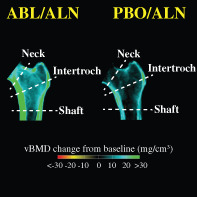

## 1 Introduction

Abaloparatide (ABL) is a bone‐forming analog of human parathyroid hormone‐related peptide (PTHrP) that is indicated for the treatment of postmenopausal women with osteoporosis (PMO) at high risk of fracture.^(^ ^1^ ^)^ In the pivotal phase 3 ACTIVE study in women with PMO, 18 months of ABL treatment increased areal bone mineral density (aBMD) at the total hip, femoral neck, and lumbar spine versus placebo, and at the total hip and femoral neck versus open‐label teriparatide, a parathyroid hormone (PTH) analog.^(^ ^2^ ^)^ The ACTIVE study also showed significant reductions in vertebral, nonvertebral, clinical, and major osteoporotic fractures with ABL versus placebo (PBO), and fewer major osteoporotic fractures with ABL versus teriparatide.

BMD gains with bone‐forming agents, including teriparatide and the sclerostin inhibitor romosozumab, are reversible after treatment cessation,^(^ ^3^, ^4^ ^)^ and the same is likely true of ABL. As such, patients who complete or discontinue bone‐forming therapy are often advised to transition to another osteoporosis therapy to prevent bone loss.^(^ ^5^ ^)^ Antiresorptive therapies are a common choice, and transitioning to alendronate (ALN) or other bisphosphonates can preserve and, in some cases, further enhance BMD gains.^(^ ^6^, ^7^, ^8^, ^9^, ^10^ ^)^ Although no head‐to‐head data are available, women with PMO who transition to alendronate after 12 to 24 months of therapy with PTH receptor agonists appear to show greater hip and spine BMD gains during treatment with antiresorptive therapy^(^ ^6^, ^7^, ^9^ ^)^ compared with those who transition to alendronate after 12 months of romosozumab therapy.^(^ ^10^ ^)^ This apparent difference may relate in part to contrasting bone turnover rates at the time of transition to antiresorptive therapy: after 12 months of PTH receptor agonist therapy, bone turnover markers are generally elevated over baseline whereas they are below baseline after 12 months of treatment with romosozumab.^(^ ^2^, ^10^, ^11^ ^)^ Higher bone turnover is associated with greater remodeling space that can rapidly refill during antiresorptive therapy,^(^ ^12^ ^)^ and bisphosphonates are preferentially taken up at sites of active bone remodeling,^(^ ^13^ ^)^ which increases their skeletal uptake^(^ ^14^ ^)^ and may enhance their efficacy.

The 24‐month ACTIVE extension study (ACTIVExtend) showed that 24 months of ALN after 18 months of ABL reduced vertebral and nonvertebral fracture risk compared with 24 months of ALN after 18 months of PBO.^(^ ^7^ ^)^ Alendronate treatment after abaloparatide led to further increases in aBMD at the total hip, femoral neck, and lumbar spine, but the effects of this treatment regimen on trochanteric regions, the femoral shaft, and cortical versus trabecular compartments have not been previously described. Localized treatment responses within the hip may influence the likelihood of fractures initiating or propagating within the affected subregion, and might also have implications for the fixation of orthopedic implants including hip prostheses.^(^ ^15^, ^16^, ^17^ ^)^

Cortical and trabecular bone parameters are typically obtained via imaging modalities that rely on 3D quantitative computed tomography (QCT)‐based technologies. Such modalities were not included in ACTIVE or ACTIVExtend, but standard two‐dimensional (2D) hip dual energy X‐ray absorptiometry (DXA) scans can now be processed using 3D‐DXA software to model cortical and trabecular measurements, with results that correlate well with QCT results.^(^ ^18^ ^)^ Previous post hoc analyses with 3D‐DXA modeling in ACTIVE indicated greater increases in cortical volumetric BMD (Ct.vBMD) of the total hip, femoral neck, trochanter, and shaft subregions after 18 months of ABL versus PBO and teriparatide.^(^ ^19^ ^)^ Estimated strength indices of the femoral neck and lower shaft, including cross‐sectional moment of inertia and section modulus, also showed greater increases after 18 months of ABL versus PBO and teriparatide. The current post hoc analyses for ACTIVExtend describe 3D‐DXA data for the total hip and hip subregions after sequential treatment comprising 18 months of ABL or PBO followed by 24 months of open‐label ALN. Correlation analyses were also performed to investigate the extent to which BMD gains with ALN during ACTIVExtend relate to levels of bone turnover markers at the time of therapeutic transition to alendronate from ABL or PBO treatment.

## 2 Patients and Methods

### 2.1 ACTIVE trial

ACTIVE was a phase 3, double‐blind, placebo‐controlled trial.^(^ ^2^ ^)^ A total of 2463 postmenopausal women aged 49 to 86 years from 10 countries were randomized 1:1:1 to receive daily subcutaneous injections of ABL (80 μg/day) or matching PBO, or open‐label teriparatide (20 μg/day), for a period of 18 months. Eligibility criteria included BMD *T*‐scores ≤ −2.5 and > −5.0 at the femoral neck or lumbar spine with either two or more mild or one moderate vertebral fractures, or a history of a low‐energy nonvertebral fracture within the past 5 years. Subjects >65 years of age were included with the above fracture criteria and a BMD *T*‐score ≤ −2.0 and > −5.0 at either site, or without meeting fracture criteria with a BMD *T*‐score ≤ −3.0 and > −5.0. The complete eligibility criteria and study protocol have been reported.^(^ ^2^ ^)^ DXA scans were collected at the hip at baseline and at months 6, 12, and 18. Serum markers of bone turnover, procollagen type I N‐terminal propeptide (s‐P1NP), and carboxy‐terminal cross‐linking telopeptide of type I collagen (s‐CTX) were measured in a subset of subjects at months 1, 3, 6, 12, and 18.

### 2.2 ACTIVExtend trial

Following the 18 months of ACTIVE and 1 month for reconsent, 558 subjects in the ABL group and 581 subjects in the PBO group completed those treatments and entered the ACTIVExtend study to receive open‐label ALN monotherapy (70 mg/week) for 24 months.^(^ ^7^ ^)^ Subjects in ACTIVExtend underwent DXA scans and serum markers of bone turnover were analyzed at months 6, 12, 18, and 24 (cumulative months 25, 31, 37, and 43).

### 2.3 3D‐DXA analyses

**Fig. 1:**
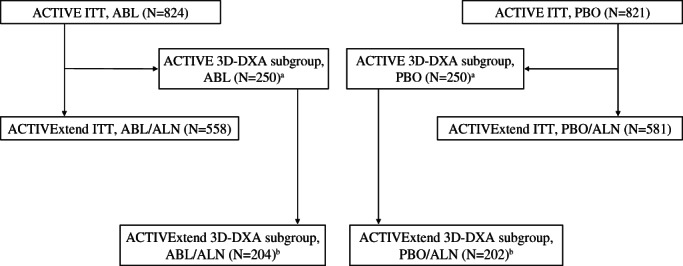
Selection of 3D‐DXA subjects from ACTIVExtend. ^a^Retrospective analysis of a subgroup of 250 randomly selected subjects from each study arm in ACTIVE. ^b^Subjects from each 3D‐DXA subgroup from ACTIVE that participated in the ACTIVExtend. Subjects were stratified by study site and subject ethnicity to ensure uniformity across the groups. ABL = abaloparatide; ALN = alendronate; DXA = dual‐energy X‐ray absorptiometry; ITT = intent to treat; PBO = placebo.

As previously reported,^(^ ^19^ ^)^ a subgroup of 250 randomly selected subjects from each study arm in ACTIVE with 3D‐DXA scans was chosen for 3D‐DXA analyses. These subjects were stratified by study site and subject ethnicity to ensure uniformity across the groups. Of the 250 subjects with DXA scans available in each treatment arm of ACTIVE, 202 subjects from the placebo group and 204 from the abaloparatide group participated in ACTIVExtend^(^ ^7^ ^)^ (Fig. 1). In this post hoc analysis, blinded DXA scans for this ACTIVExtend subgroup, performed at months 6 and 18 in ACTIVE and month 43 of ACTIVExtend, retrospectively underwent 3D modeling via 3D‐SHAPER® (v2.10.1; Galgo Medical, Barcelona, Spain).

**Fig. 2:**
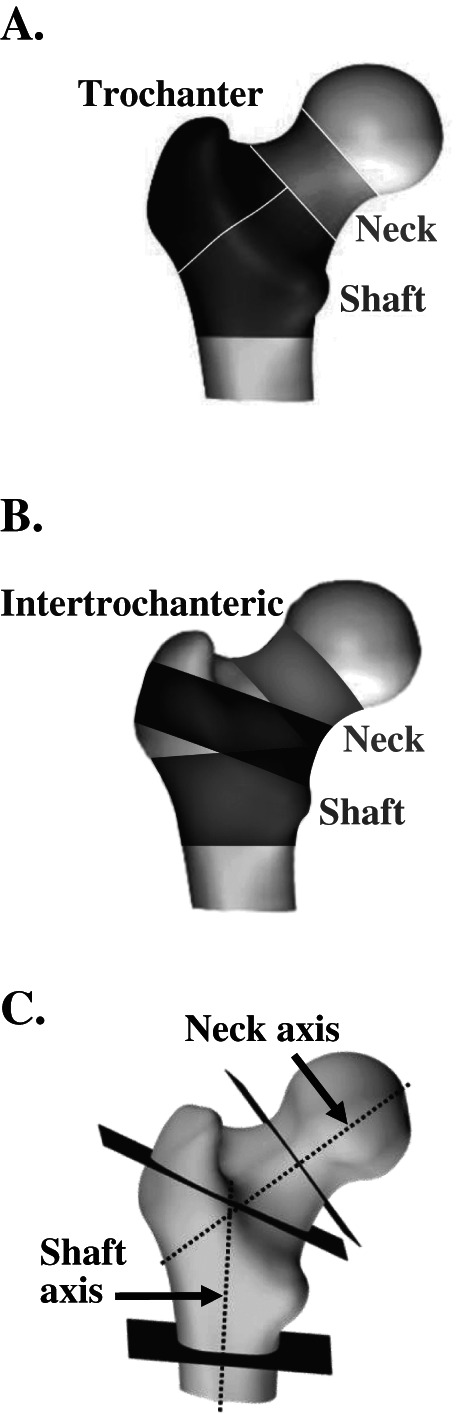
(*A*) Regions of interest for BMC, vBMD, and integral, trabecular, and cortical region volumes. (*B*) Subregions of interest for Ct.Th and Ct.sBMD. (*C*) Bending axes and section slices for hip structural measurements (CSMI, section modulus, buckling ratio) at the neck, the intertrochanteric region and the lower shaft. BMC = bone mineral content; BMD = bone mineral density; CSMI = cross‐sectional moment of inertia; Ct.sBMD = cortical surface BMD; Ct.Th = cortical thickness; vBMD = volumetric BMD.

DXA‐based 3D modeling provides a QCT‐like characterization of the trabecular and cortical components of the proximal femur. The process by which the patient‐specific 3D models are generated from 2D‐DXA images, using a population‐based model and for segmentation of the cortical bone, has been previously described.^(^ ^18^ ^)^ Cortical bone was segmented by fitting a function of location of the cortex, Ct.vBMD (mg/cm^3^), cortical thickness (Ct.Th; mm), imaging blur, and density of surrounding tissues to the density profile calculated along the normal vector at each vertex of the proximal femur surface mesh.^(^ ^20^ ^)^ Changes from baseline were calculated for Ct.Th, trabecular and cortical vBMD in mg/cm^3^, and cortical surface BMD (Ct.sBMD; product of Ct.Th and Ct.vBMD) in mg/cm^2^ for the total hip region and hip subregions (Fig. 2A‐C). Estimated strength indices comprising cross‐sectional moment of inertia, section modulus, and buckling ratio were calculated for the neck, intertrochanteric, and lower shaft subregions using methods previously described.^(^ ^21^ ^)^ Section modulus was calculated as cross‐sectional moment of inertia divided by the maximum distance (*d*_max_) between the center of mass and periosteal surface. Buckling ratio was calculated as *d*_max_ divided by mean Ct.Th.

### 2.4 Statistical analyses

Baseline comparisons were expressed in percentages or absolute values and comparisons were made by paired *t* tests. Treatment group comparisons were based on a mixed‐effect repeated measures model adjusted for body mass index (BMI), age, value at baseline, DXA scanner model, treatment group, and visit interaction. Correlation analyses were performed in a subset of the ACTIVExtend 3D‐DXA population for whom serum bone turnover marker data were generated. Spearman coefficients (*r*) were generated within each treatment group between serum biomarkers of bone turnover at the end of ACTIVE and percent change in 2D‐DXA and 3D‐DXA parameters during ACTIVExtend (months 18 to 43). All statistical analyses were performed within SAS version 9.4 (SAS Institute Inc., Cary, NC, USA). 3D spatial distribution of mean vBMD changes were evaluated between baseline and the different follow‐up points. All inferential tests were two‐tailed and statistical significance was set at 5%.

### 2.5 Ethics approval

The ACTIVE and ACTIVExtend studies were approved by the appropriate ethics committees and internal review boards at participating institutions and conducted in accordance with the recommendations of the Declaration of Helsinki in its revised edition (Tokyo, 2004) and the guidelines for current Good Clinical Practice (GCP).

## 3 Results

Subject characteristics of the 3D‐SHAPER subpopulation at baseline were well matched between groups (no significant differences) (Table 1), and were consistent with the overall baseline characteristics of the larger ACTIVE and ACTIVExtend populations, as well as with the subjects from ACTIVE included in a previous post hoc 3D‐DXA analysis.^(^ ^2^, ^7^, ^19^ ^)^

**Table 1:** Baseline Characteristics for 3D‐DXA Subgroup in ACTIVExtend

Characteristic	PBO/ALN (*n* = 202)	ABL/ALN (*n* = 204)
Age (years)		
Mean (SD)	68.8 (5.82)	68.4 (6.42)
Median (min, max)	69.0 (55, 86)	68.0 (50, 84)
BMD *T*‐score, mean (SD)		
Lumbar spine	−2.9 (0.8)	−2.9 (0.9)
Total hip	−1.9 (0.7)	−1.9 (0.7)
Femoral neck	−2.2 (0.7)	−2.1 (0.7)
Baseline prevalent vertebral fracture, *n* (%)	50 (24.9)	44 (21.6)
≥1 prior nonvertebral fracture within past 5 years, *n* (%)	51 (25.2)	55 (27.0)
No prior fracture, *n* (%)	86 (42.6)	75 (36.8)

ABL = abaloparatide; ALN = alendronate; DXA = dual‐energy X‐ray absorptiometry; PBO = placebo; SD = standard deviation.

### 3.1 Areal BMD by 2D‐DXA

**Fig. 3:**
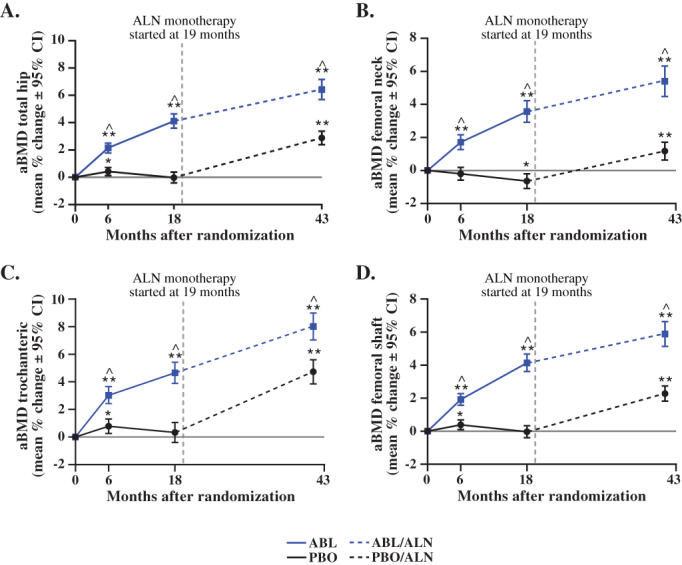
aBMD by 2D‐DXA for the 3D‐DXA Subpopulation in ACTIVE and ACTIVExtend. (*A*) aBMD total hip (mean % change ± 95% CI). (*B*) aBMD femoral neck (mean % change ± 95% CI). (*C*) aBMD trochanteric (mean % change ± 95% CI). (*D*) aBMD femoral shaft (mean % change ± 95% CI). *p < 0.01; **p < 0.0001 vs baseline; ^*p* < 0.0001 vs PBO or PBO/ALN. ABL = abaloparatide; aBMD = areal bone mineral density; CI = confidence interval; DXA, dual energy X‐ray absorptiometry; PBO = placebo.

From month 0 to month 43, total hip and femoral neck aBMD for the 3D‐DXA subpopulation increased in both treatment groups, with larger gains in those who received ABL followed by ALN (ABL/ALN) compared with those who received PBO followed by ALN (PBO/ALN) (total hip, 6.4% versus 2.9%; femoral neck, 5.4% versus 1.2%, respectively). These findings are consistent with treatment effects demonstrated in the overall ACTIVExtend population (Fig. 3A‐D).^(^ ^7^ ^)^ Increases in aBMD of the trochanter and shaft were also greater at month 43 in the ABL/ALN group than the PBO/ALN group (trochanter, 8.0% versus 4.7%; shaft, 5.9% versus 2.3%).

### 3.2 3D‐DXA parameters

Baseline bone mineral content (BMC) values (shown in Table S1) indicate the relative balance of cortical versus trabecular bone for the total hip and hip subregions at the beginning of ACTIVE (month 0) and ACTIVExtend (month 18) based on 3D‐DXA analysis. At month 0, cortical bone in the PBO and ABL groups comprised approximately 63% of total hip BMC, 54% of femoral neck BMC, 53% of trochanter BMC, and 72% of shaft BMC. Cortical, trabecular, and integral BMC of the total hip and all three subregions increased significantly from baseline to 18 months in the ABL/ALN group compared with PBO/ALN (*p* < 0.0001 for all).

By 3D‐DXA, trabecular vBMD (Tb.vBMD) of the total hip and all three subregions increased from month 0 to month 18 in the abaloparatide group but not in the placebo group. Tb.vBMD increased from month 0 to month 43 in both treatment groups, with greater increases in the ABL/ALN group versus PBO/ALN (Table 2).

**Table 2:** Mean Percent Change (95% CI) in Trabecular vBMD of the Total Hip and Hip Subregions by 3D‐DXA

	Baseline (month 0) to month 18			Baseline (month 0) to month 43		
Location	ABL (*n* = 204)	PBO (*n* = 202)	ABL versus PBO	ABL/ALN (*n* = 204)	PBO/ALN (*n* = 202)	ABL/ALN versus PBO/ALN
Total hip	8.77^a^ (7.59, 9.95)	−0.14 (−1.16, 0.88)	9.01^b^ (7.49, 10.53)	10.87^a^ (9.38,12.35)	4.30^a^ (3.11, 5.49)	6.66^b^ (4.83, 8.50)
Femoral neck	8.98^a^ (7.75, 10.21)	−0.02 (−1.11, 1.07)	9.13^b^ (7.52, 10.73)	11.03^a^ (9.46, 12.60)	3.16^a^ (2.04, 4.28)	8.00^b^ (6.12, 9.88)
Trochanter	9.76^a^ (8.08, 11.45)	0.48 (−1.17, 2.13)	9.54^b^ (7.29, 11.79)	14.08^a^ (11.98, 16.17)	7.59^a^ (5.62, 9.55)	6.75^b^ (4.06, 9.43)
Shaft	13.75^a^ (10.32, 17.17)	−0.26 (−1.58, 1.05)	13.94^b^ (10.42, 17.46)	12.37^a^ (8.96, 15.78)	4.52^a^ (2.83, 6.20)	7.78^b^ (3.96, 11.60)

ABL = abaloparatide; ALN = alendronate; CI = confidence interval; DXA = dual energy X‐ray absorptiometry; PBO = placebo; vBMD = volumetric bone mineral density.

^a^ *p* < 0.0001 versus baseline.

^b^ *p* < 0.0001 between groups.

**Fig. 4:**
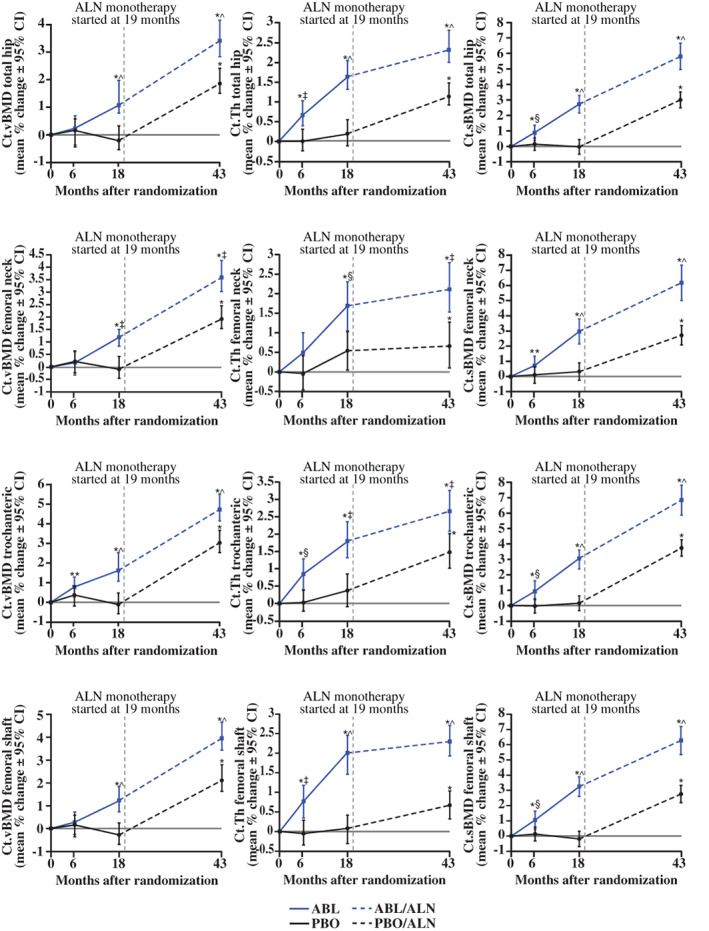
Subregion 3D‐DXA modeling cortical endpoints. *p < 0.001; **p < 0.05 versus baseline; ^*p* < 0.001; ‡*p* < 0.01; §*p* < 0.05 versus PBO or PBO/ALN. ABL = abaloparatide; ALN = alendronate; BMD = bone mineral density; Ct.sBMD = cortical surface BMD; Ct.Th = cortical thickness; Ct.vBMD = cortical volumetric BMD; DXA = dual‐energy X‐ray absorptiometry; PBO = placebo.

**Fig. 5:**
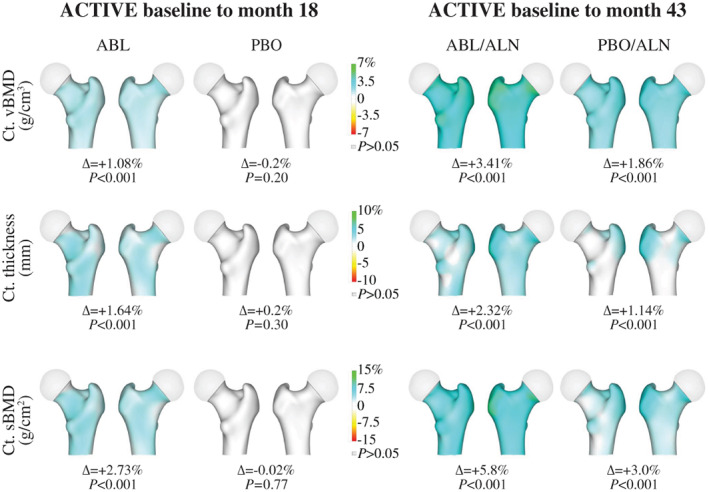
3D‐DXA images reflecting changes in Ct.sBMD, Ct.vBMD, and Ct.Th. Data represent mean absolute differences within each group relative to baseline. Increases from baseline in Ct.sBMD, Ct.vBMD, and Ct.Th are presented in blue‐green color while decreases are presented in yellow‐red colors. ABL = abaloparatide; ALN = alendronate; BMD = bone mineral density; Ct.sBMD = cortical surface BMD; Ct.Th = cortical thickness; Ct.vBMD = cortical volumetric BMD; DXA, dual energy X‐ray absorptiometry; PBO = placebo.

For 3D‐DXA cortical parameters, increases from month 0 to month 43 were significant for the total hip in both groups (*p* < 0.001) and were greater with ABL/ALN versus PBO/ALN at 43 months (Ct.Th, 2.32% versus 1.14%; Ct.vBMD, 3.41% versus 1.86%; Ct.sBMD, 5.82% versus 3.0%, respectively; all *p* < 0.001). Similarly, Ct.Th, Ct.vBMD, and Ct.sBMD of hip subregions increased from month 0 to month 43 in both groups (all *p* < 0.001 versus baseline), with greater increases at month 43 in the ABL/ALN group versus PBO/ALN (all *p* < 0.01) (Figs. 4 and 5 and Table S2).

**Fig. 6:**
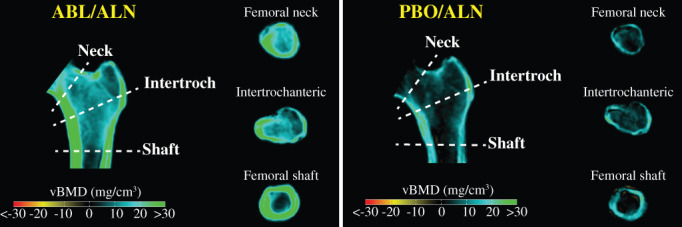
Cross‐sectional views of mean vBMD changes from baseline to month 43. Mid‐coronal and cross‐sectional slices indicate group mean changes in vBMD from baseline to 43 months. Blue‐green regions reflect increases from baseline in vBMD, black regions reflect no change, and yellow‐red regions indicate reduced vBMD. ABL = abaloparatide; ALN = alendronate; vBMD = volumetric bone mineral density; DXA = dual‐energy X‐ray absorptiometry; PBO = placebo.

In Fig. 6, mid‐coronal sections depicting mean changes in vBMD indicate greater increases in cortical regions of the ABL/ALN versus PBO/ALN groups, as reflected by more green color in the former versus latter group. Inner regions representing the medullary/cancellous compartment also show greater vBMD gains with ABL/ALN versus PBO/ALN as represented by relatively greater blue color with ABL/ALN. These differences are also apparent in cross‐sectional slices through each femur subregion.

### 3.3 Bone structural parameters

**Fig. 7:**
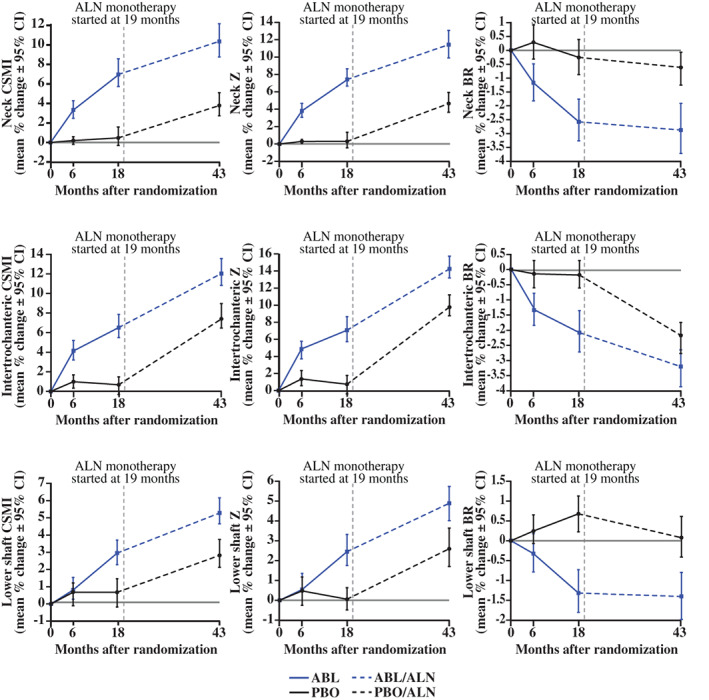
Mean (95% CI) changes in biomechanical parameters of hip subregions. ABL = abaloparatide; ALN = alendronate; BR = buckling ratio; CI = confidence interval; CSMI = cross‐sectional moment of inertia; PBO = placebo; Z = section modulus. For ABL/ALN versus BL, all *p* < 0.0001 except lower shaft CSMI at 6 months (*p* < 0.05), lower shaft Z at 6 months (*p* = not significant), and lower shaft BR at 6 months (*p* = not significant); for ABL/ALN versus PBO/ALN, all *p* < 0.05 except lower shaft at 6 months (*p* = not significant). *n* = 202 for PBO/ALN and *n* = 204 for ABL/ALN.

The ABL/ALN group showed greater improvements in bone structural parameters that are considered relevant to bone strength. Cross‐sectional moment of inertia, section modulus, and buckling ratio were significantly increased at 43 months for all three subregions versus baseline (all *p* < 0.0001) and versus PBO/ALN (all *p* < 0.02) (Fig. 7).

### 3.4 Serum markers of Bone turnover

Data for serum markers of bone turnover were available for 145 subjects in the PBO/ALN group and 154 in the ABL/ALN group with 2D‐DXA measurements, and for a smaller subset (PBO/ALN, *n* = 62; ABL/ALN, *n* = 64) with 3D‐DXA measurements. For the ABL/ALN group, P1NP at the end of ACTIVE (month 18) correlated with percent change in all DXA and 3D‐DXA parameters during ACTIVExtend (months 18 to 43), with *r* ranging from 0.341 to 0.419 (*p* = 0.000–0.006) (Table 3 and Fig. S1). CTX at the end of ACTIVE also correlated with percent change in all DXA and 3D‐DXA parameters for the ABL/ALN group, with *r* ranging from 0.285 to 0.457 (*p* = 0.000–0.004). Significant correlations between month 18 bone turnover markers and DXA and 3D‐DXA parameters during ACTIVE were not consistently observed in the PBO/ALN group (data not shown).

**Table 3:** Correlation Analyses for ABL/ALN Treatment Group (*n* = 64) of Serum P1NP and CTX Levels at Month 18 versus Percent Change in 2D‐DXA and 3D‐DXA Parameters During ACTIVExtend (Months 18 to 43)

2D‐DXA and 3D‐DXA parameters (% change from month 18 to 43)	Bone turnover marker (month 18)	*r* (Spearman)	*p*
Total hip aBMD	P1NP	0.549	<0.001
	CTX	0.538	<0.001
Femoral neck aBMD	P1NP	0.413	<0.001
	CTX	0.367	0.003
Total hip Ct.vBMD	P1NP	0.352	0.004
	CTX	0.384	0.002
Total hip Tb.vBMD	P1NP	0.341	0.006
	CTX	0.400	0.001
Total hip Int.vBMD	P1NP	0.416	0.001
	CTX	0.457	<0.001
Total hip Ct.sBMD	P1NP	0.379	0.002
	CTX	0.355	0.004

ABL = abaloparatide; ALN = alendronate; aBMD = areal BMD; BMD = bone mineral density; Ct.vBMD = cortical volumetric BMD; CTX = carboxy‐terminal cross‐linking telopeptide of type I collagen; DXA = dual‐energy X‐ray absorptiometry; Int.vBMD = integral volumetric BMD; P1NP = procollagen type I N‐terminal propeptide; PBO = placebo; sBMD = surface BMD; Tb.vBMD = trabecular volumetric BMD.

## 4 Discussion

The current analyses from the 3D‐DXA subpopulation of ACTIVE and ACTIVExtend indicate that sequential osteoporosis therapy comprising 18 months of ABL followed by 24 months of ALN significantly increases aBMD of the total hip and hip subregions versus treatment with PBO followed by ALN. These subgroup findings corroborate similar positive effects of ABL followed by ALN on total hip and femoral neck aBMD in the overall ACTIVE and ACTIVExtend populations^(^ ^7^ ^)^ while further indicating favorable trochanter and shaft aBMD responses.

aBMD gains with ALN at the total hip and hip subregions were generally similar in the 3D‐DXA subpopulations of both treatment groups, indicating that the therapeutic effects of ALN on aBMD are largely preserved when administered after 18 months of ABL. The magnitude of total hip and hip subregion aBMD gains with ALN after ABL are notable in light of the substantial aBMD gains that occurred with prior ABL treatment. In contrast, a recent study in women with PMO indicated that greater gains in total hip and femoral neck aBMD during 18 to 24 months of teriparatide therapy were associated with lesser aBMD gains after switching to 12 months of follow‐on therapy comprising oral or intravenous bisphosphonates or denosumab.^(^ ^8^ ^)^ Subjects receiving teriparatide during ACTIVE were not eligible to participate in ACTIVExtend, and the lack of other studies directly comparing BMD responses to follow‐on osteoporosis therapy after ABL versus teriparatide leaves open the question of whether these two bone‐forming agents differentially influence responses to subsequent therapy with ALN or other antiresorptive agents.

Beyond new evidence that alendronate increases aBMD of the total hip and all hip subregions when administered after ABL, the current study's main focus was to expand on previous 3D‐DXA results for ABL treatment in ACTIVE to understand hip subregional, cortical, and trabecular changes with ALN during ACTIVExtend. 3D‐DXA assessments of cortical and trabecular BMC at the ACTIVE baseline indicate that the total hip and hip subregions are composed primarily of cortical bone, with trabecular bone making up the remaining 36%, 46%, 47%, and 28% of BMC for the total hip, femoral neck, trochanter, and shaft subregions, respectively. At baseline of ACTIVExtend, the ABL/ALN group exhibited significant increases in trabecular, cortical, and integral BMC of the total hip and all hip subregions compared with the PBO/ALN group. During ACTIVExtend, sequential ABL/ALN or PBO/ALN treatment increased Ct.Th, Ct.vBMD, Ct.sBMD, and Tb.vBMD of the total hip and all three hip subregions, and also improved estimated strength indices of hip subregions, with greater improvements at month 43 with ABL/ALN versus PBO/ALN.

Average gains from baseline in Tb.vBMD after 43 months of sequential ABL/ALN therapy ranged from 10.9% to 14.1% for the total hip and hip subregions, and most of these gains manifested during the ABL treatment phase, with modest additional increases during ALN follow‐on therapy in all regions. Gains in Ct.Th with the ABL/ALN regimen also manifested primarily during the ABL treatment phase, with lower rates of increase during ALN follow‐on therapy. These results are consistent with the hypothesis that increases in Tb.vBMD and Ct.Th during ABL treatment reflect increases in bone volume, with follow‐on ALN therapy potentially contributing to modest additional volume gains through the closure of remodeling space. Though undocumented in the current study, increased matrix mineralization during ALN therapy may also have contributed to Tb.vBMD gains, a mechanism that could also lead to modest increases in Ct.Th through partial volume effects.^(^ ^22^ ^)^

Gains in Ct.vBMD with the sequential ABL/ALN regimen ranged from 3.4% to 4.7% for the total hip and hip subregions, with the rate of Ct.vBMD gains generally similar during the ABL and ALN treatment phases. Ct.sBMD, the product of Ct.vBMD and Ct.Th, showed greater increases over 43 months in the ABL/ALN versus PBO/ALN group for the total hip and all hip subregions, with the rate of Ct.sBMD gains in the ABL/ALN group roughly similar before and after therapeutic transition to ALN. Mechanistic hypotheses for ALN effects on Ct.vBMD include increased matrix mineralization and closure of intracortical and endocortical remodeling spaces.

Several studies summarized by Cosman and colleagues^(^ ^23^ ^)^ describe BMD responses in individuals with osteoporosis who transitioned from bisphosphonate therapy to PTHR1 agonists, a treatment sequence paradigm that is increasingly recognized as being suboptimal for maximizing BMD gains. The current study is among the few describing BMD responses after transitioning from PTHR1 agonists to bisphosphonates, a regimen that aligns with newer treatment guidelines supporting frontline use of anabolic agents in patients with very high fracture risk.^(^ ^24^ ^)^ These results from ACTIVExtend support the treatment sequence of PTHR1 agonists followed by potent bisphosphonate therapy, as do the results of a therapeutic transition study showing robust total hip, femoral neck, and lumbar spine aBMD gains in women with PMO after transitioning from PTH(1‐84) therapy to ALN.^(^ ^6^ ^)^

The mechanism of action for bisphosphonate therapy involves their preferential adsorption to exposed bone mineral, the extent of which increases with bone resorption and bone formation. A bone‐scanning study in women with PMO showed that teriparatide increases skeletal uptake of a radiolabeled diagnostic bisphosphonate, providing a hypothetical mechanism by which prior therapy with PTHR1 agonists may prime the skeleton to respond to therapeutic bisphosphonates.^(^ ^14^ ^)^ Bisphosphonates also increase BMD by allowing closure of remodeling space, the extent of which tends to increase during treatment with PTHR1 agonists. In apparent contrast to the robust BMD responses to ALN after ABL, women with PMO who transitioned from 1 year of romosozumab to 2 years of ALN therapy exhibited minimal BMD gains after the transition, which may relate in part to lower bone formation and resorption markers after 12 months of romosozumab.^(^ ^10^ ^)^ Based on these observations, we performed exploratory correlation analyses of serum levels of bone turnover markers at the ACTIVExtend baseline versus BMD changes during ACTIVExtend to test the hypothesis that higher bone turnover at the time of ALN treatment initiation is associated with greater subsequent BMD gains within each treatment group. All correlations were highly significant for the ABL/ALN group (*p* < 0.0001), which supports our hypothesis but does not indicate whether these relationships are driven by differences in skeletal alendronate uptake, the extent of remodeling space and closure thereof, or other factors. A study in women with PMO indicated that s‐P1NP levels after 18 to 24 months of teriparatide therapy correlated positively with change in lumbar spine aBMD after transition to 12 months of oral or intravenous bisphosphonates or denosumab, but such relationships were not significant for the total hip or femoral neck.^(^ ^8^ ^)^ This lack of significance could relate, at least in part, to the inclusion of subjects who received risedronate, a bisphosphonate that causes lesser total hip and femoral neck BMD gains compared with ALN.^(^ ^25^ ^)^

One limitation of these correlation analyses is the 1‐month delay between month 18 bone turnover marker evaluations and the initiation of ALN treatment while subjects underwent reconsent to enter ACTIVExtend. The effect of this brief treatment gap on these relationships is unclear but is probably more likely to have reduced than increased *r* values. Other study limitations include the newness of 3D‐Shaper technology and its reliance on QCT scans from a largely white population to develop population‐based hip reference models. Implications of the current results in terms of hip fracture risk also remain unclear. Results for the overall ACTIVE and ACTIVExtend study populations showed that five subjects in the PBO/ALN group had an incident hip fracture versus no subjects in the ABL/ALN group (nominal *p* = 0.03),^(^ ^7^ ^)^ and the current 3D‐DXA data show favorable effects of ABL/ALN versus PBO/ALN on estimated biomechanical parameters at the total hip and hip subregions. However, it is beyond the scope of the current analyses to understand which, if any, 3D‐DXA parameters may have contributed to these apparent differences in fracture rate.

## 5 Conclusion

These results, which are among the first to describe skeletal responses to ALN after treatment with a PTHR1 agonist that is currently approved for the treatment of PMO, indicate significant improvements in aBMD, Ct.vBMD, and Tb.vBMD of the total hip and hip subregions with ABL/ALN versus PBO/ALN. For most 2D‐DXA and 3D‐DXA parameters, particularly the cortical measures, ALN effects were relatively similar whether ALN was administered after ABL or after PBO. These results support the use of abaloparatide as the initial treatment in sequential therapy with ALN in women with PMO at very high fracture risk, a treatment paradigm consistent with recently updated clinical practice guidelines from organizations such as American Association of Clinical Endocrinology (AACE) and European Society for Clinical and Economic Aspects of Osteoporosis, Osteoarthritis and Musculoskeletal Diseases (ESCEO).^(^ ^24^, ^26^ ^)^

## Conflict of Interest

RW and LH are employees of Galgo Medical and paid consultants of Radius Health, Inc.; LH is a shareholder of Galgo Medical. PJK, JB, and YW are employees and shareholders of Radius Health, Inc.

### Peer Review

The peer review history for this article is available at https://publons.com/publon/10.1002/jbm4.10612.

## Supporting information


**Appendix S1:** Supporting information

## Data Availability

Data that underlie the results reported in a published article may be requested for further research 6 months after completion of US Food and Drug Administration (FDA) or European Medicines Agency (EMA) regulatory review of a marketing application (if applicable) or 18 months after trial completion (whichever is latest). Radius will review requests individually to determine whether (i) the requests are legitimate and relevant and meet sound scientific research principles, and (ii) are within the scope of the subjects' informed consent. Prior to making data available, requestors will be required to agree in writing to certain obligations, including without limitation, compliance with applicable privacy and other laws and regulations. Proposals should be directed to info@radiuspharm.com.
