# Supplementary material for: Proximal Femur Responses to Sequential Therapy With Abaloparatide Followed by Alendronate in Postmenopausal Women With Osteoporosis by 3D Modeling of Hip Dual‐Energy X‐Ray Absorptiometry (DXA)
Source: JBMR Plus. 2022 Mar 10;6(4):e10612. doi: 10.1002/jbm4.10612 (PMC9009108; doi:10.1002/jbm4.10612)
Supplement: Supplementary file 1 — Appendix S1: Supporting information [file JBM4-6-e10612-s001.docx]

**Supplemental Material**

**Supplemental Table 1. Baseline Mean (±SD) BMC Results by 3D-DXA**

|  | **ACTIVE baseline (month 0)** | | **ACTIVExtend baseline  (month 18)** | | |
| --- | --- | --- | --- | --- | --- |
|  | **PBO (N=202)** | **ABL  (N=204)** | **PBO/ALN (N=202)** | **ABL/ALN (N=204)** | ***P* value for % change from month 0 to month 18 for PBO/ALN vs ABL/ALN** |
| Total hip |  |  |  |  |  |
| Integral (g) | 17.23 ± 3.97 | 17.40 ± 3.93 | 17.21 ± 3.95 | 18.46 ± 4.20 | <0.0001 |
| Cortical (g) | 10.94 ± 2.27 | 11.10 ± 2.28 | 10.95 ± 2.27 | 11.50 ± 2.39 | <0.0001 |
| Trabecular (g) | 6.28 ± 1.83 | 6.30 ± 1.83 | 6.26 ± 1.83 | 6.96 ± 1.98 | <0.0001 |
| Femoral neck |  |  |  |  |  |
| Integral (g) | 3.26 ± 0.74 | 3.28 ± 0.72 | 3.26 ± 0.74 | 3.50 ± 0.81 | <0.0001 |
| Cortical (g) | 1.77 ± 0.40 | 1.79 ± 0.36 | 1.78 ± 0.40 | 1.86 ± 0.40 | <0.0001 |
| Trabecular (g) | 1.49 ± 0.38 | 1.49 ± 0.40 | 1.49 ± 0.38 | 1.64 ± 0.45 | <0.0001 |
| Trochanter |  |  |  |  |  |
| Integral (g) | 4.75 ± 1.25 | 4.79 ± 1.28 | 4.74 ± 1.23 | 5.11 ± 1.35 | <0.0001 |
| Cortical (g) | 2.51 ± 0.57 | 2.55 ± 0.63 | 2.51 ± 0.56 | 2.65 ± 0.65 | <0.0001 |
| Trabecular (g) | 2.24 ± 0.74 | 2.25 ± 0.73 | 2.23 ± 0.73 | 2.46 ± 0.76 | <0.0001 |
| Femoral shaft |  |  |  |  |  |
| Integral (g) | 9.22 ± 2.11 | 9.32 ± 2.08 | 9.21 ± 2.11 | 9.86 ± 2.21 | <0.0001 |
| Cortical (g) | 6.66 ± 1.41 | 6.76 ± 1.41 | 6.66 ± 1.41 | 7.00 ± 1.46 | <0.0001 |
| Trabecular (g) | 2.55 ± 0.79 | 2.56 ± 0.80 | 2.55 ± 0.79 | 2.86 ± 0.87 | <0.0001 |

ABL = Abaloparatide; ALN = alendronate; BMC = bone mineral content; PBO = placebo.

**Supplemental Table 2. Mean Percent Change (95% CI) in Ct.sBMD (mg/cm^2^) of the Total Hip and Hip Subregions by 3D-DXA**

|  | Baseline to month 18 | | | Baseline to month 43 | | |
| --- | --- | --- | --- | --- | --- | --- |
|  | ABL  (N=204) | PBO (N=202) | ABL vs PBO difference in LSM | ABL/ALN^a^ (N=204) | PBO/ALN (N=202) | ABL/ALN vs PBO/ALN difference in LSM |
| Total hip | 2.73^a^ (2.16, 3.29) | −0.02 (−0.49, 0.44) | 2.76 ^b^ (2.03, 3.49) | 5.82^a^ (4.97, 6.68) | 3.00^a^ (2.49, 3.51) | 2.84^b^ (1.86, 3.81) |
| Femoral neck | 2.97^a^ (2.15, 3.79) | 0.33 (−0.23, 0.90) | 2.64^b^ (1.64, 3.64) | 6.18 ^a^ (5.01, 7.34) | 2.72^a^ (2.09, 3.36) | 3.46^b^ (2.14, 4.77) |
| Intertrochanteric | 3.09^a^ (2.39, 3.78) | 0.15 (−0.40, 0.69) | 2.95^b^ (2.07, 3.83) | 6.80^a^ (5.77, 7.83) | 3.71^a^ (3.11, 4.30) | 3.10^b^ (1.94, 4.26) |
| Femoral shaft | 3.25^a^ (2.61, 3.89) | −0.19 (−0.70, 0.32) | 3.44^b^ (2.63, 4.26) | 6.28^a^ (5.35, 7.20) | 2.76^a^ (2.19, 3.33) | 3.52^b^ (2.46, 4.57) |

ABL = abaloparatide; ALN = alendronate; BMC = bone mineral content; BMD = bone mineral density; CI = confidence interval; Ct.sBMD = cortical surface BMD; LSM, least squares mean; PBO = placebo.

^a^ *P*<0.0001 vs baseline.

^b^ *P*<0.0001 between groups.

**Supplemental Figure 1. Correlation Analyses for ABL/ALN Treatment Group (n=64) of Serum PINP and CTX Levels at Month 18 Versus Percent Change in Total Hip aBMD During ACTIVExtend (Months 18-43)**

1. **PINP**


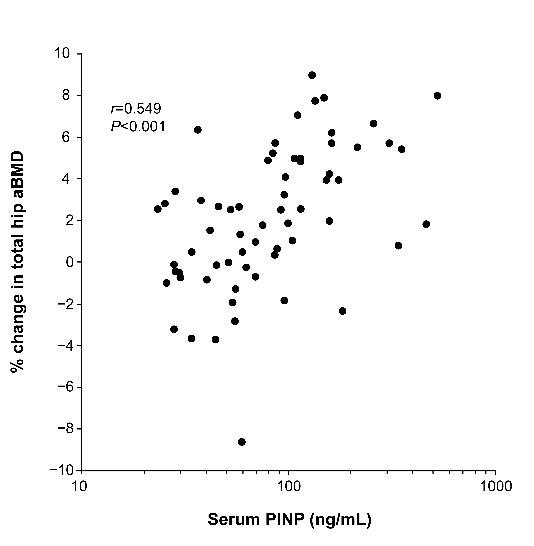


1. **CTX**


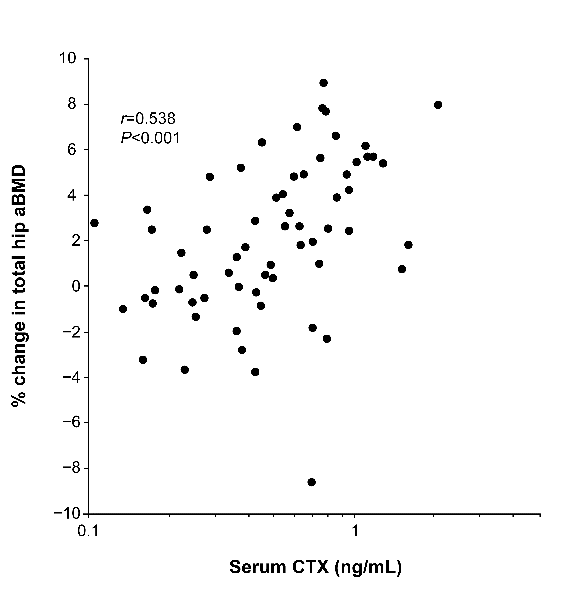


ABL = abaloparatide; aBMD = areal bone mineral density; ALN = alendronate; CTX = carboxy-terminal cross-linking telopeptide of type I collagen; PINP = procollagen type I N-terminal propeptide.
